# Analysis of Heat‐Mass Transfer and Exergy in Heating Process of Chilled Ready‐to‐Eat Foods Using High‐Power Microwave Ovens

**DOI:** 10.1155/ijfo/4410623

**Published:** 2026-04-30

**Authors:** Jirapol Klinbun, Phadungsak Rattanadecho, Waraporn Klinbun

**Affiliations:** ^1^ Department of Mechanical Engineering, Faculty of Engineering, Rajamangala University of Technology Krungthep, Bangkok, Thailand, rmutk.ac.th; ^2^ Department of Mechanical Engineering, Faculty of Engineering, Thammasat University (Rangsit Campus), Pathum Thani, Thailand, tu.ac.th

**Keywords:** chilled ready-to-eat foods, exergy efficiency, microwave heating, moisture distributions, multi compartment plastic tray

## Abstract

The objective of this research was to develop a 3‐D coupled electromagnetics, heat transfer, and mass transfer model for transient microwave heating of chilled ready‐to‐eat (RTE) foods. The effect of power level, multi compartment plastic tray design, and placement angle on the temperature and moisture distributions and the rate of heating were predicted. Then, the energy and the exergy in microwave heating system were analyzed in detail. The model was validated for heating 150 g of steamed rice and 80 g of steamed chicken breast within multi compartment plastic tray for 60 s in 800 W microwave oven on a no turntable. The spatial variation of the top surface temperatures of the chilled RTE foods were captured by an infrared camera, the transient temperatures at eight locations recorded using k type thermocouple sensors. The temperature distribution during heating all showed good agreement with the simulation results. The difference rate of temperature rise (RTR) values between experimental and simulation data were 0.40°C/s–0.57°C/s (for steamed rice) and 0.07°C/s–0.42°C/s (for steamed chicken breast). Results illustrated that the developed model comprehensively explained the phenomena during the microwave heating of chilled RTE foods. The optimum study conditions had the maximum heating efficiency of 1300 W, foods in tray A1, and placement angle of 135°. Additionally, it was found that the multi compartment tray design becomes the most influencing factor to increase exergy efficiency. These findings will contribute to the development of more efficient heating protocols and improved design of microwave heating systems for RTE food applications.

## 1. Introduction

The growing demand for ready‐to‐eat (RTE) meals has revolutionized modern eating habits. Microwave heating is the primary method used to heat these foods [1]. High‐power microwave ovens have gained importance both in homes and commercially due to their fast‐heating capabilities. However, the complex interaction between electromagnetic waves makes the task of uniform temperature distribution and optimization of food quality very challenging [1]. Heat transfer and mass phenomena during microwave heating of food, especially chilled RTEs, occur simultaneously, such as absorption of electromagnetic energy, thermal conductivity, moisture migration, and phase change [2]. An insight into the coupled interaction mechanism has been developed to maximize the rate and uniformity of the heating process while assuring safety without a loss of its nutritional value and sensory qualities [1]. Previous research has looked at the effects of several factors, including packaging [3–14], nutrition [15–17], microbes [18–21], convenience [22], and speed [23–27]. This research includes both experimentation and numerical modeling, with recent advances focusing on precision control systems, smart packaging integration, and real‐time monitoring capabilities. For example, in heat‐mass transfer studies in microwave applications, Klinbun and Rattanadecho [28] have conducted experiments using different microwave power levels to evaluate their impact on heating performance. It has been observed that higher power levels generally result in higher heating rates but may also lead to more significant temperature variations within the food. Bhattacharya and Basak [10] have designed containers with compartments to assess the heating rate and non‐uniformity. It was found that the shape of the container, whether round or square, can influence the distribution of microwave energy. Liu et al. [8] confirmed that cubic samples had better power absorption capability, but spherical samples showed better temperature distribution uniformity. Furthermore, the orientation of the food sample inside the oven can result in variations in the electromagnetic field distribution and temperature uniformity. Zhang et al. [29] discovered that near the edge or center of the turntable, which can impact the heating rate and uniformity, it is important to consider both the shape and orientation of the food sample to achieve optimal temperature uniformity in microwave heating. Moreover, in exergy analysis applications in food processing, energy analysis provides information on thermodynamic efficiency and energy consumption during heating, which is relevant due to the importance of energy efficiency in food processing [30–37]. The application of thermodynamic analysis has become known as an increasingly common approach for the design and optimization of thermal systems [38]. The first law of thermodynamics pertains to the preservation of energy inside a system through its transformation from one form to another. It does not give any information on the reversibility features of thermodynamic processes. The study of energy availability in a process may be conducted using the second law. Examples of past research, Darvishi et al. [39] found that energy and exergy efficiency, drying kinetics, diffusivity of moisture, and activation energy are affected by moisture content, slice thickness, and microwave power. These parameters are crucial for optimizing the drying process and ultimately enhancing the efficiency of kiwi slice drying. Prommas et al. [30] carried out an energy and exergy analysis of drying process of non‐hygroscopic porous packed bed using multi‐feed microwave convective air and the continuous belt system. The researchers found that the energy utilization ratio and exergy efficiency are dependent on factors such as particle size, hot air temperature, and magnetron location. Even though there has been a lot of research on microwave heating, we still need in‐depth studies that combine heat and mass transfer with energy efficiency, particularly for high‐power uses involving chilled RTE foods. Current literature lacks comprehensive exergy analysis specifically tailored to high‐power microwave applications for chilled RTE foods. Additionally, there is limited research on the optimization of process parameters considering both energy efficiency and food quality simultaneously. The integration of heat‐mass transfer modeling with thermodynamic analysis remains underexplored in the context of commercial food processing applications. This study aims to fill this knowledge gap by addressing three main research questions: (1) investigate the coupled mechanisms of electromagnetic energy absorption, thermal conductivity, moisture migration, and phase change processes under high‐power conditions; (2) determine the quality of energy utilization and identify sources of irreversibility in the heating process; and (3) establish optimal operating conditions considering microwave power, container design, and food placement for maximum energy efficiency while maintaining food quality.

The objective of this study is to develop a mathematical model to calculate exergy, second‐law efficiency, heat and mass transfer, and energy during the microwave heating process of chilled RTE foods. The effects of microwave power (800, 1000, and 1300 W), multi compartment plastic tray design (circle, semicircle), and placement angle (0°, 45°, 90°, and 135°) are investigated. Steamed chicken breast and steamed rice are sample. This research makes several significant contributions to the field: (1) this study provides the first detailed thermodynamic analysis specifically designed for high‐power microwave applications in chilled RTE food processing, filling a critical gap in the literature; (2) the research develops a unified mathematical framework that combines heat and mass transfer phenomena with thermodynamic analysis, providing a more complete understanding of the heating process; and (3) the study delivers actionable recommendations for optimal process parameters that can be directly implemented in commercial food processing operations, bridging the gap between theoretical research and practical application. These studies provide valuable insights for both consumers and the food business. Consumers can learn how to use the microwave in a variety of environments to cook a wide range of pre‐packaged foods. Consequently, this results in the utilization of energy in an effective manner. The efficacy of microwave technology in reducing the duration and energy consumption required for heating has been well established. The industry can identify the optimal packing material by conducting an in‐depth exploration of this field.

## 2. Materials and Methods

### 2.1. Samples Preparation and Physical Model

**Figure 1:**
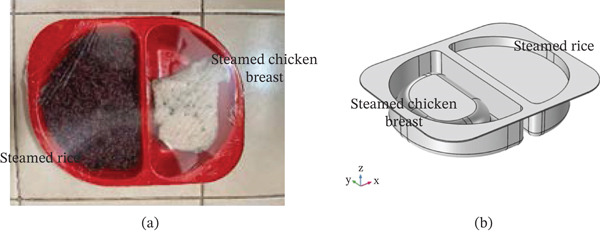
(a) Sample used in experiment, (b) physical model used in numerical simulation.

Steamed chicken breast and steamed rice were purchased from a local market in Bangkok, Thailand as seen in Figure 1a. It was stored at refrigeration conditions (7°C) for experimental trials. In total, 150 g of steamed rice consisted of 6 g of protein, 3 g of fat, 65 g of carbohydrate, and 10 mg of sodium and has an energy value of 311 kcal. While in total, 80 g of steamed chicken consisted of 18 g of protein, 1 g of fat, 1 g of carbohydrate, and 420 mg of sodium and has an energy value of 90 kcal. These nutritional facts were used to calculate dielectric and thermal values. Before the heating experiments, steamed chicken breast of similar sizes were selected, with an average diameter value of approximately 7 cm. The sample were separated into three groups. Figure 1b shows physical model used in numerical simulation. Three main elements were present as steamed rice, steamed chicken, and multi‐part container. This physical model provided a basis for conducting numerical simulations pertaining to the thermal effects of RTE foods under the effect of electromagnetic waves.

### 2.2. Material Properties

#### 2.2.1. Dielectric Properties

In this study, open‐ended coaxial‐line probes (Püschner GmbK+Co.KG, France) have been used successfully for convenient broad‐band permittivity measurements on RTE foods. The testing frequencies were ranged from 300 MHz to 3.0 GHz. The precision was not more than 2% of the dielectric constant (*ε* ^′^) and 5% of the dielectric loss factor (*ε* ^′′^). Before testing, the instrument was calibrated by the dielectric constant measurements of air, short circuit, and pure water at 25°C. The sample was positioned in a wide glass tube, and the open coaxial probe was inserted into the tube. The sample holder was immersed in a temperature‐regulated water bath. The testing temperature ranges were specified from 0°C to 70°C. The dielectric constants of the sample were measured every 5°C until 70°C. The experiments were repeated three times. The results were reported of the relative complex permittivity as a function of temperature and at 2.45 GHz. The typical error of the dielectric properties measurements was approximately 5% following standard calibration procedures. Table 1 shows the value of the relative complex permittivity (*ε* = *ε* ^′^ − *j* *ε* ^′′^), loss tangent (tan*δ*), and penetration depth (*d*_*p*_) obtained from measurements and previous literature [40, 41], respectively. All three properties have consistent relationship characteristics. It can be noted that the steamed chicken can generate internal heat better than the steamed rice because the loss tangent of the steamed chicken was higher than that of the steamed rice. Finally, the penetration depths were obtained from the dielectric properties that were in the ranges of 8.45–11.35 mm (for steamed chicken) and 7.88–10.52 mm (for steamed rice).

**Table 1:** Material properties of the steamed chicken breast and the steamed rice at 7°C.

Properties	Data				
	Unit	Steamed chicken breast		Steamed rice	
		Measured	Previous published	Measured	Previous published
Relative complex permitivity, (*ε*)	[—]	66.75—j16.94	37.84—j14.42	61.87—j19.32	48.34—j17.46
Loss tangent, (tan*δ*)	[—]	0.253	0.381	0.312	0.361
penetration Depth, (*d*_*p*_)	[mm]	9.47	8.45	8.02	7.88
Density, (*ρ*)	[kg/m^3^]	1066	1037	1203	1050
Specific heat, (*C*_*p*_)	[J/kg·K]	3608	3521	2913	2930
Thermal conductivity, (*k*)	[W/m·K]	0.406	0.509	0.339	0.694

#### 2.2.2. Thermal Properties

This work obtained the thermal properties (thermal conductivity (*k*) and specific heat capacity (*C*_*p*_)) of RTE food using composition data and temperature‐dependent mathematical models of the various meal components [42, 43]. The mathematical models predicted the thermal properties of food components as functions of temperature in the range of –40°C–150°C. Then, it compared the thermal properties of steamed chicken breast and steamed rice at 7°C with previous research results [44, 45] as shown in Table 1.

### 2.3. Experimental Setup

The experimental microwave heating system consisted of a high wattage microwave oven (NE 1356, Panasonic) with features of 220 V, 50 Hz, and a frequency of 2450 MHz. The dimensions of the microwave cavity were 330 × 310 × 175 mm (18 L) Heating experiments were carried out at three microwave power levels: 800, 1000, and 1300 W. The K‐type thermocouples were sensors that measure temperature, and the thermal image was captured immediately after the heating was finished using an IR camera (SC640, accuracy ±2°C, 640 × 480 pixels, FLIR Systems, Boston, MA). Three replications of each experiment were performed according to a preset microwave output power and time schedule, and the data given were an average of these results. To study the heating profile of RTE foods within the various multiple‐compartment container styles (A1, A2, A3), in different placement angle the microwave oven (L1, L2, L3, L4), the experimental procedures were followed: (1) the samples were filled in the container, and the initial temperature was measured. (2) The sample was placed in the center of the microwave cavity. (3) Microwave oven was turned on for 60 s. (4) When the experiment was finished, the temperature was measured and captured. The three multi‐compartment tray designs investigated were defined as follows: (i) Tray A1—both compartments semicircular; (ii) Tray A2—both compartments rectangular; (iii) Tray A3—one circular and one rectangular compartment. The four placement positions were defined as the angle between the long axis of the food tray and the waveguide aperture: L1 (0°), L2 (45°), L3 (90°), and L4 (135°). Eight 0.5 mm diameter, type‐K thermocouples were inserted into the food samples. For steamed rice, four thermocouples were positioned at defined x–y coordinates covering the center and edge regions, with probe tips at mid‐depth (z = 15 mm). For the chicken breast, four thermocouples were inserted along the major axis at equally‐spaced intervals, tips at the geometric center of thickness.

### 2.4. Mathematical Model

A mathematical method has been developed for analyzing the behavior of microwave foods by solving the unsteady state heat transfer differential equations. The model was utilized for extensive systems where Maxwell’s equations is applicable. It considered changeable thermal, moisture, and electromagnetic characteristics. The numerical solution was formulated utilizing a finite element technique for three‐dimensional systems implemented in COMSOL Multiphysics 5.3 (COMSOL AB, Stockholm, Sweden), combining the RF module, heat transfer module, and transport of diluted species module.

#### 2.4.1. Electromagnetic Field

As a dielectric material, limestone will not conduct free charges (*σ* = 0) and interactions with the magnetic field are negligible. Thus, the following Maxwell’s equation was solved to determine the distribution of the electric field in a microwave cavity [9]:

(1) ∇×μr−1∇×E?−k02εrE?=0

where *ε*_r_ = (*ε*/*ε*_0_)Œ°_*r*_, Œ*μ*_*r*_ is the relative dielectric permittivity,*μ*_r_ = *μ*/*μ*_0_, Œ°_*r*_, Œ*μ*_*r*_is the relative magnetic permeability, *k*_0_ is the complex valued wavenumber in vacuum, *ε*_0_ = 8.854 × 10^−12^ F/m and *μ*_0_ = 4*π* × 10^−7^ H/m are the permeability and permittivity properties of vacuum, and $\overset{?}{E}$ is the electric field intensity (V/m).

#### 2.4.2. Heat Transfer

To describe heat transfer within the food, the volumetric power generation is formulated, which includes the internal generation term originated by the energy delivered by microwaves [9]:

(2) ρCp∂T∂t=k∇2T+Qabs

where $œ\overset{{}^{\circ}}{A},C_{p}œ\overset{{}^{\circ}}{A},C_{p}\rho ,C_{P},$ k are the density (kg/m3), specific heat (J/kg·°C), thermal conductivity (W/m·°C) of food, respectively. T is the temperature (°C).  *Q*_*a* *b* *s*_ is the volumetric heat generation term (W/m3) originated by the interaction with microwaves which represents the average power loss associated with the electric and magnetic fields developed within the material [9]:

(3) Qabs=2πf∙ε0∙εr"∙E?2

where *f* is the frequency (Hz).

#### 2.4.3. Moisture Transfer

The heat equation coupled the moisture diffusion inside the sample (steamed rice and steamed chicken breast). The water evaporated using all the absorbed energy when the internal temperature reached the boiling point. The evaporation rate of the water was obtained [11]:

(4) ∂Cw∂t=∇∙Dw∇Cw+Rw

where *C*_*w*_ is the water concentration (kg/m3) and R_w_ = *Q*/*L*, R_W_ = *Q*_*a* *b* *s*_/*L*, is the evaporation rate (kg/s m3). The evaporation rate was determined on the premise that, after the boiling point is attained, all the heat generated is utilized solely for the purpose of evaporating water, if there is water ready for evaporation. *L* = 2260(*k* *J*/*k* *g*) is the latent heat of water for evaporation.

The initial and boundary conditions are the following:

(5) t=00 T=Ti ≤t≤L

(6) Cw=Cw,i

(7) t>00 −k∂T∂t=hT−T∞+Lkm′Cw,s−Cequi x= and x=L

(8) −Dw∂C∂x=km′Cw,s−Cequi

#### 2.4.4. Exergy Analysis

In the scope of the second law of exergy analysis, it helps find places and reasons for performance loss, offering valuable insights for enhancing system efficiency. The general exergy balance in microwave cavity was expressed as follows [31, 34]:

(9) Exin=Exabs+Exref+Extra⏟≅Exloss

The input exergy of the microwave heating and drying can be determined by

(10) Pin×t?Exin=m×ex2−m×ex1+exevap.×t?exergy evaporation?Exabs+Exref+Extra⏟≅Exloss

The rate of exergy transfer due to evaporation in the cavity was:

(11) ex′evap=1−T0Tp×mwv.λwp

(12) mwv.=mt+Δt−mtΔt

The specific exergy (J/s) can be calculated by

(13) ex=CpT−T0−T0lnTT0

The reference of state conditions considered is: *T*_0_ = 25^°^C, *P*_0_ = 101.325 kPa.

The exergy efficiency of the microwave heating and drying can be estimated by

(14) ηex=100×ExabsExin

The specific exergy loss can be determined as follows

(15) Exloss=Exin−Exabsmwv

**Figure 2:**
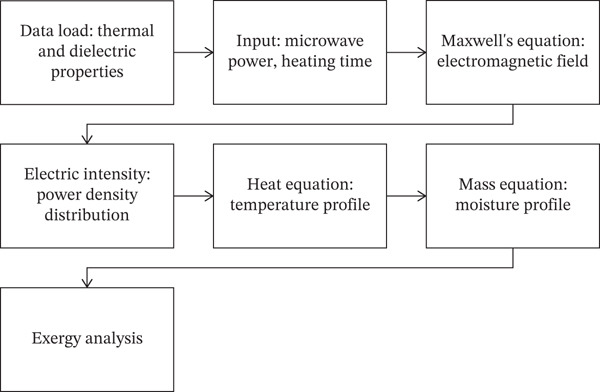
Computational schemes.

In Figure 2, the mathematical model was created to predict the electromagnetic distribution inside the oven, the absorbed microwave power, temperature profile, and mass transfer inside the RTE foods during the microwave heating process. After that, the second law of thermodynamics was considered as the potential of producing useful work to obtain the exergy transfer.

### 2.5. Mesh Independence Study

Tetrahedral mesh was used for the sample, container, and microwave oven. A total of five mesh levels were assessed: normal (588,289 elements), fine, finer (1,024,360 elements), extra fine, and extremely fine (7,001,728 elements). The convergence criterion was defined as a relative change in mean temperature of less than 0.1% between successive refinement levels. For each mesh level, the mean and maximum temperature in steamed chicken and steamed rice at t = 60 s was recorded. Results confirm that the “finer” mesh (1,024,360 elements) satisfies this criterion, with a maximum relative deviation of 0.07% in chicken temperature and 0.002% in rice temperature compared with the extremely fine mesh. The calculation time ratio (finer: extremely fine ≈1:2.1) justifies the selection of the finer mesh for all subsequent simulations, balancing accuracy and computational efficiency.

## 3. Results and Discussion

### 3.1. Model Verification

**Figure 3:**
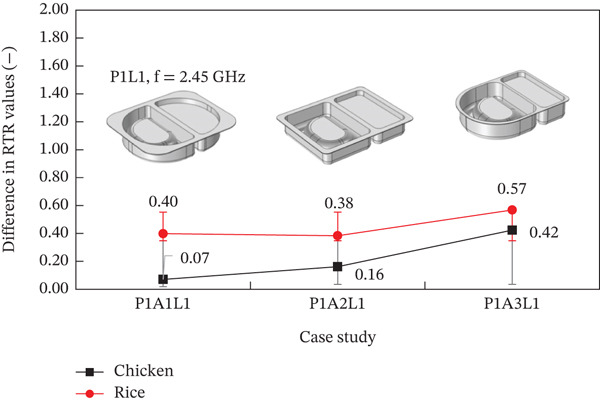
Validation of the model by analyzing the difference in RTR values.

In this research, tetrahedral mesh was used for the sample, container, and microwave oven. A total of five grid types were used: normal, fine, finer, extra fine, and extremely fine. Normal grid was the least number of elements (equal 588,289), while extremely fine grid was the highest number of grids (equal 7,001,728). From the results, it was found that the temperature inside the steamed chicken breast of each grid type had a different value at the third decimal place, while the temperature inside the steamed rice of each grid type had a different value at the fifth decimal place. However, when considering the calculation time, it was found that in the cases of extra fine and extremely fine, the calculation time was twice as much as in the cases of normal, fine, and finer. Therefore, this research chose to use finer element size with a grid number of at least one million to save computation time. In addition, the RTR (Rate of Temperature Rise, °C/s) obtained in the present study and those obtained from experiment were compared based on the same geometric model. The RTR value was calculated from heating time, initial, and final temperatures [46]. The temperature value was obtained from the measurement of 8‐type K thermocouples (with an accuracy of ±0.1) inserted throughout the area of the steamed rice and steamed chicken breast. For the validate test, the microwave power was 800 W (P1), and the tray placement was at an angle of 0° with the waveguide (L1). Figure 3 shows that the difference in RTR values for steamed chicken was not significantly different; they were significantly different for each package of steamed rice. This is due to the influence of the shape of the container.

### 3.2. Effect of Microwave Power

**Figure 4:**
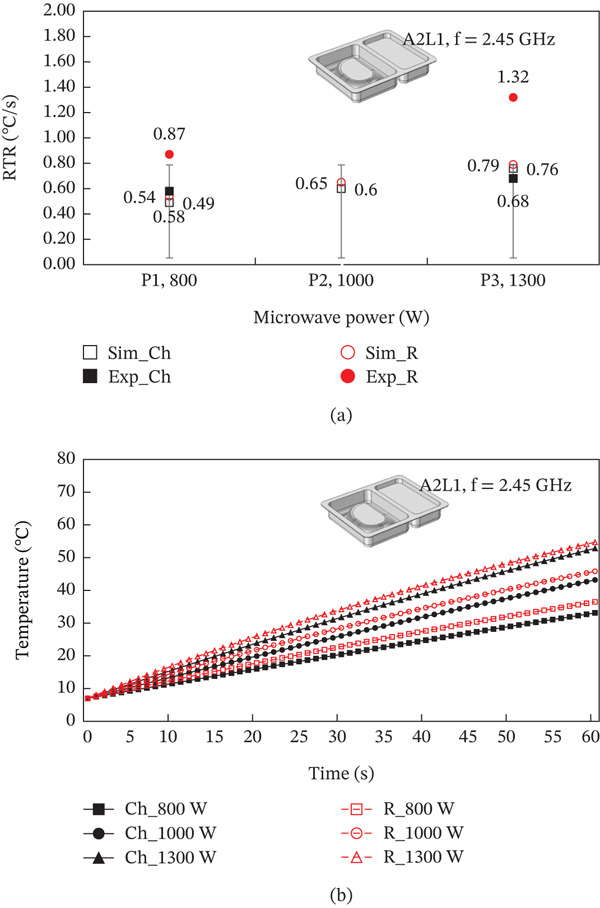
Influence of microwave power on the (a) RTR value (b) temperature value inside steamed chicken and steamed rice.

**Figure 5:**
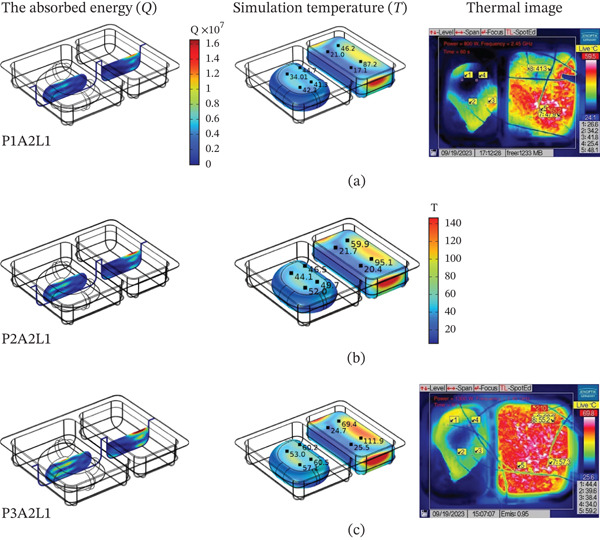
The energy absorbed and temperature distribution pattern (simulation and thermal image) inside RTE foods at the following power: (a) 800 W, (b) 1000 W, and (c) 1300 W.

The microwave power was 800, 1000, and 1300 W by using the food tray style A2 (both parts were rectangular in shape) and the food tray placement position L1 (at an angle of 0 degrees with the waveguide) as a case study. From Figure 4a, the RTR value of the steamed chicken and the steamed rice was used to compare the predicted and experimental values of temperature. In steamed rice, the experimentally determined RTR values were higher than predicted by the model. In the case of steamed chicken, the RTR values obtained from experiments and calculations were close to each other. Figure 4b shows the temperature increasing with time. The sample had the highest temperature at a high wattage of 1300 W because it has more energy and heats up faster [47, 48]. Figure 5 illustrates the energy absorbed and temperature distribution pattern (simulation and thermal image) within the RTE foods.

**Figure 6:**
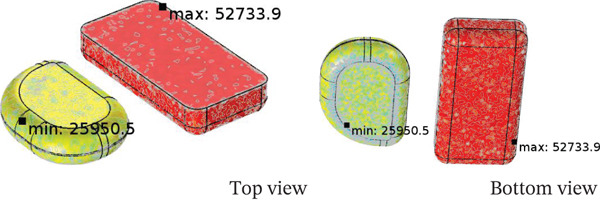
Moisture transport inside steamed chicken and steamed rice in case of P3A2L1.

**Figure 7:**
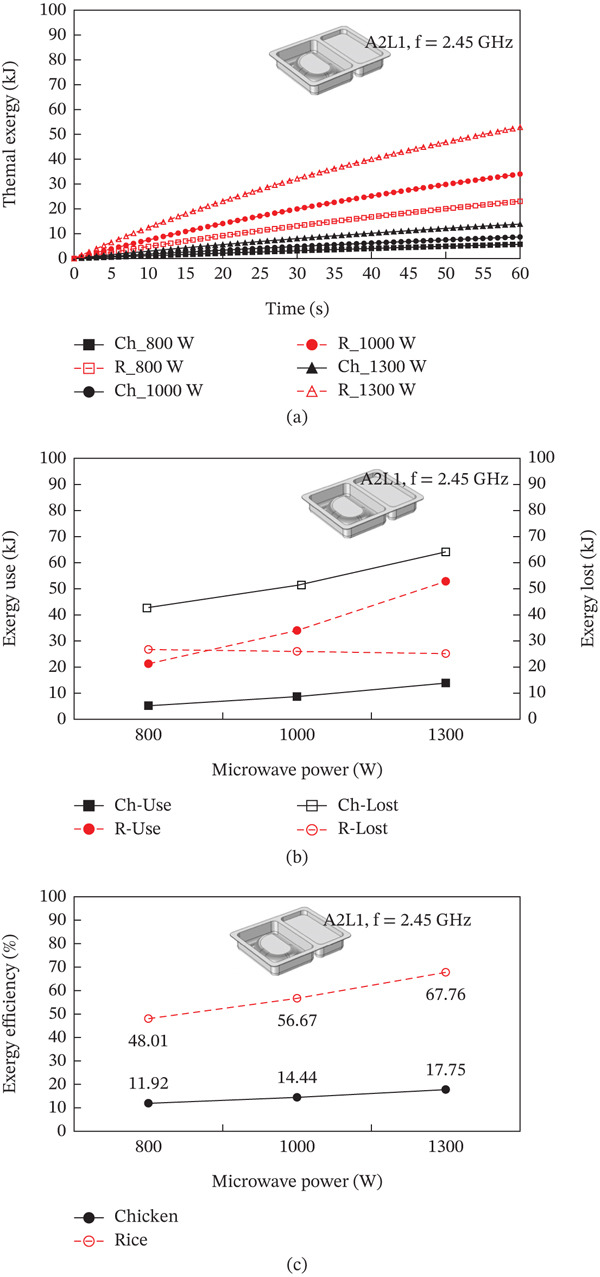
Influence of microwave power on (a) the exergy transfer, (b) the exergy utilization, and (c) the exergy efficiency of steamed chicken and steamed rice.

The heating pattern of temperature serves as a key indicator as to the physics behind microwave heating and its methods of dielectric heating. At the 2.45 GHz frequency, an electromagnetic field interacts with polar molecules (mostly water), which leads to volumetric heating because of the rapid forceful oscillation of molecules, this occurs instead of surface conduction heating. The differences in heating rates between the steamed chicken and rice are a result of their varied dielectric properties, moisture content, and distribution patterns. In the steamed chicken analysis, the close agreement between experimental and predicted RTR values (Figure 4a) confirms that the dense protein matrix of chicken breast yields uniform dielectric properties. Additionally, the chicken’s fibrous muscle tissue aids in the appropriate convective heat transfer Figure 6). Rather large amounts of moisture (> 65%–75%) makes consistent microwave absorption possible throughout the sample volume. In the case of steamed rice, the higher RTR values obtained through experimentation suggests a greater disparity than predicted. Because of the heterogeneous structure of rice, it can be subjected to uneven heating. The process of gelatinization of starches within each rice grain leads to moisture content that is highly variable between the surfaces of the grain and the insides. This effect causes the rice grain to contain localized hot spots. In combination, such phenomena cause considerable acceleration in heating compared to the so‐called ‘typical’ heating. Figure 7 shows a strong correlation between thermal exergy and heating time at various microwave power levels. Power‐dependent exergy efficiency illustrates how good the thermodynamics of microwave heating systems are. At an 800 W input, the microwave conversion efficiencies of 10.77% for chicken and 44.32% for rice show how the energy provided does not exceed useful thermal work outcomes. Efficiency improvements with increasing power (1000 and 1300 W) suggest that higher levels of electromagnetic field intensity overcome molecular motion activation energy much better. The exergy analysis shows that high power levels result in more favorable rates of entropy generation for food matrix systems. For steamed rice, the maximum thermal exergy at 1300 W (Figure 7a) reflects the ideal values of electromagnetic field depth penetration resulting in the maximal active heating zone volume with minimal boundary losses. Spatial temperature uniformity impacts food safety and sensory quality; however, the hot and cold spots (Figure 5) suggest areas of under‐processing that could be risk given microbial safety. The surface roughness causing temperature variation indicates that food shape and geometry needs optimization to minimize temperature discrepancies during heating while preserving nutrition. Cui et al. [49] investigated microwave heating at 250–350 W and found the highest exergy transfer efficiency was at 300 W, which contrasts our findings that used 800 W as a baseline. Their results buttress our observation that energy use aggravates the need for power optimization. Temperature distribution patterns in their study appeared to be more non‐uniform than what we anticipated based on our theory of power dependent field intensity. The exergy analysis methodology employed in this study agrees with the framework set by Ranjbaran and Zare [31] regarding the processing of foods. However, in the case of rice heating, our findings indicate that we have much higher exergy efficiency values.

All the above aspects indicate that further developments for industrial applications should focus on energy consumption optimization because the exposed efficiencies at the range of 1000–1300 W can be useful for industrial microwave foods heating applications. The consistent temperature distribution patterns across power levels indicate that the heating mechanism can be scaled to larger volumes, which is important for industrial heating. Industrial advantages would be gained from the following: (1) multi‐magnetron systems with field uniformity for large processing chambers. (2) Continuous conveyor‐based production designed for optimal established power levels—and (3) thermal imaging‐based systems for quality control to supervise temperature uniformity.

To summarize, the microwaves heating optimization demonstrates an increase in energy efficiency while improving temperature uniformity and food safety relative to other methods. More so, the comprehensive exergy analysis offers a systematized approach for industrial scaling while the exhaustive literature review confirms the optimized parameters within these findings. Their implementation in commercial food processing operations promote economic viability, and for the public, safety values.

### 3.3. Effect of Food Tray Design

**Figure 8:**
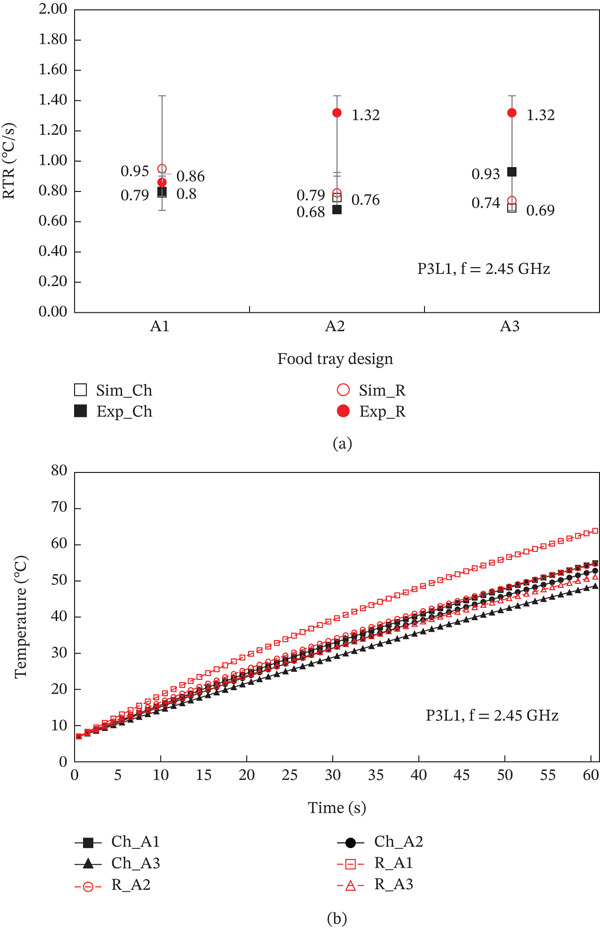
Influence of tray styles on the (a) RTR value, (b) temperature value inside steamed chicken and steamed rice.

**Figure 9:**
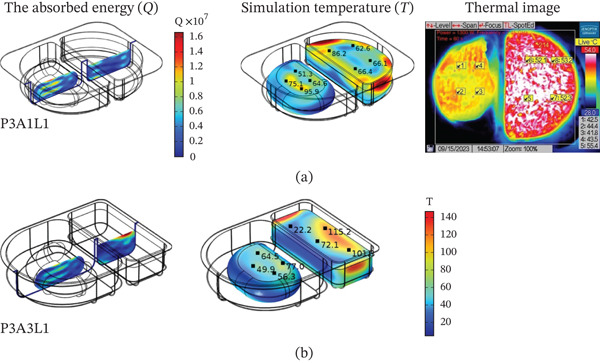
The energy absorbed and temperature distribution pattern (simulation and thermal image) inside RTE foods at the following food tray format: (a) A1 and (b) A3.

**Figure 10:**
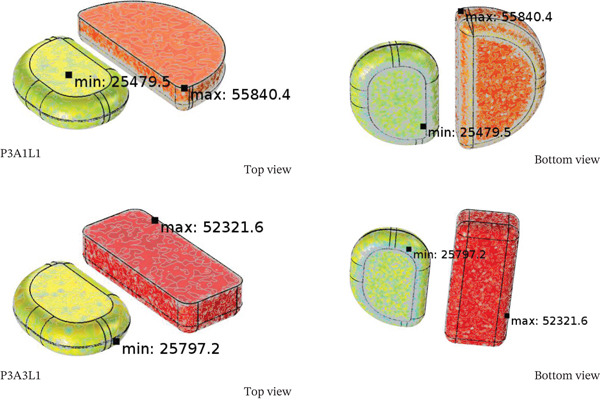
Moisture transport inside steamed chicken and steamed rice in cases of various food tray designs.

The tray styles were A1 (both semicircular shapes), A2 (both square shapes), and A3 (round and square shapes). The microwave power of 1300 W and the location of the food tray L1 (at an angle of 0° to the waveguide) were selected as a case study. Figure 8 shows (a) the RTR and (b) the patterns of temperature distribution within various food trays (A1, A2, A3). This figure represents the outcome of both calculations and experimental findings. Figure 9 demonstrates how the design of the food tray influences the energy distribution pattern within the RTE food. Figure 10 reveals a relationship between the design of the food tray and the distribution of moisture.

The A1 tray design (semicircular shapes) is superior based on electromagnetic field theory and thermal dynamics. The semicircular geometry optimally focuses fields by minimizing scattering and reflection losses of electromagnetic waves. Constructive interference patterns are created that are more efficient at concentrating microwave energy within the food volume than rectangular (A2) or mixed geometries (A3) because the semicircular shape in A1 trays functions as natural waveguide resonators that establish standing wave patterns for energy absorption. The sharp corners that curved surfaces eliminate hot spots and discontinuities fields. It thus explains why A1 attained uniform temperature distribution of 45°C–60°C over both compartments. The A2 tray with square geometry features the highest moisture distribution rates (5.3448 mol m−3 s−1 for chicken), however suffers from corner heating effects where electromagnetic energy concentrates at geometric discontinuities. This phenomenon surpasses temperature uniformity but improves moisture redistribution due to enhanced convective heat transfer within the food matrix. The interaction of tray geometry with moisture distribution reveals boundary layer dynamics are critical. Square geometries (A2) generate multiple thermal boundary layers enhancing pressure‐driven moisture migration. The generation of local velocity gradients at rectangular corners drives vapor transport from the internal skin to the surface regions, thus giving rise to the increased moisture distribution rate.

**Figure 11:**
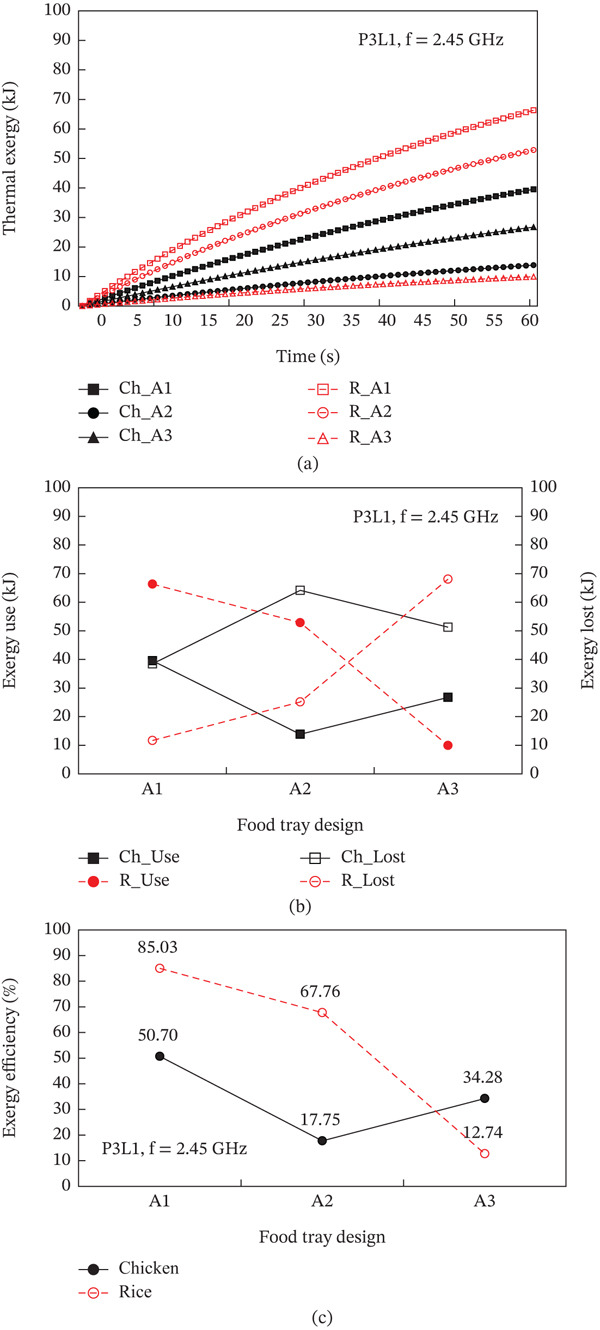
Influence of food tray design on (a) the exergy transfer, (b) the exergy utilization, and (c) the exergy efficiency of steamed chicken and steamed rice.

In point of relationship between tray design and exergy efficiency, Figure 11 shows the interaction effect of the design of the food tray on the thermal exergy that occurs during the microwave heating process. The exergy analysis reveals that geometric optimization has a direct influence on thermodynamic efficiency based on the minimization of entropy generation. The A1 tray shows the highest exergy utilization with 85.0% for rice compared to 12.74% for A3, indicating better than average conversion of electromagnetic energy to thermal energy with low irreversible loss. The design A1 semicircle leads to an almost laminar flow of energy, which decreases turbulence, and the mixing entropy associated with it. The reduced smooth gradient of energy not only decreases thermal stress on the food matrix but also makes heat transfer more efficient. On the contrary, A3 mixed geometry of circular and square shapes contains “energy dissipating” zones where the conversion of electromagnetic energy to thermal energy becomes inefficient due to geometric discontinuities. The large exergy losses of A3 design (68.06 kJ for rice) suggests strong irreversible processes at the interface of different geometric sections. Such losses lead to local overheating and non‐uniform temperature gradients with the internal thermal stress induced moisture loss that does not aid in useful heating. Moreover, the remarkable nutritionally safe and food‐preserving functions of the uniform temperature distribution because of A1 tray design are crucial. This range prevents proteins from excessive heating during inactivation of pathogens and nutrient degradation while providing adequate to inactivate them, which is maintained at the consistent temperature range of 45°C–60°C.

Microwave food packaging needs to be carefully studied to balance efficiency and food safety. The works of [50, 51] highlighted the necessity of the appropriate design and materials selection for microwave food packaging materials, ensuring they withstand microwave radiation without compromising the food’s quality. Our experimental findings confirm their assumptions, applying A1 semicircular design provided better field distribution and energy absorption. Therefore, the comprehensive review on food tray design impacts on microwave heating suggests that the geometry of the food tray is among the key factors determining energy efficiency, temperature uniformity, and food quality optimization. A1 semicircular design outperforms all others with 85.0% exergy efficiency and uniform temperature distribution. The findings are ready for immediate industrial application as they significantly reduce energy costs while enhancing food safety. The analysis done in conjunction with other studies highlights the uniqueness of this method of geometrical optimization and its ability to transform commercial food processing systems.

### 3.4. Effect of Sample Placement

**Figure 12:**
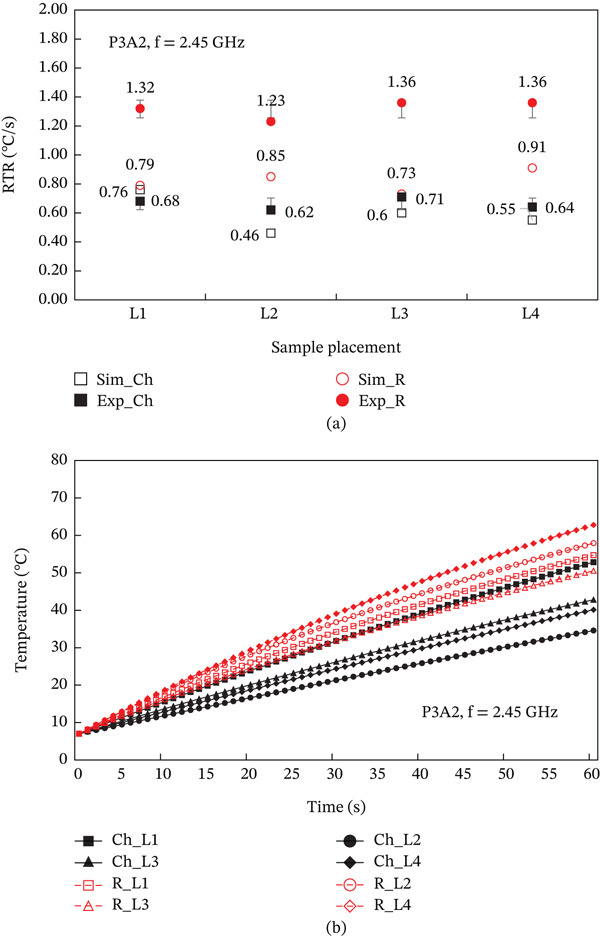
Influence of sample placement on the (a) RTR value, (b) temperature value inside steamed chicken and steamed rice.

**Figure 13:**
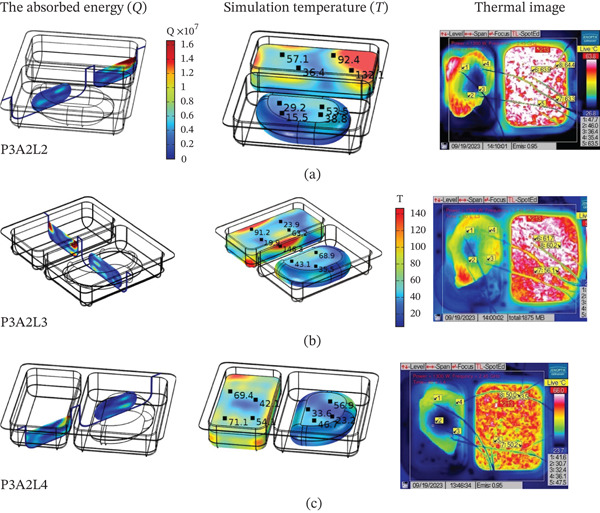
The energy absorbed and temperature distribution pattern (simulation and thermal image) inside RTE foods at the following positions: (a) L2, (b) L3, and (c) L4.

**Figure 14:**
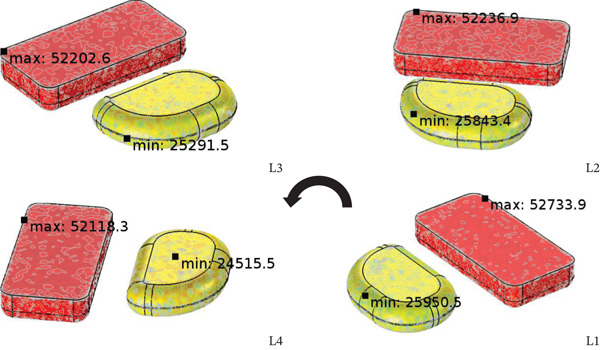
Moisture transport inside steamed chicken and steamed rice in cases of various sample placement.

The position of placing the food tray at an angle to the waveguide was 0, 45, 90, 135 degrees (L1, L2, L3, L4) by choosing a microwave wattage equal to 1300 W and food tray format A2 (both parts are rectangular shape) as a case study. Figures 12, 13 and 14 show what happens to the energy absorbed, the pattern of temperature distribution (simulation and thermal image), and the transfer of moisture in the RTE food when the food tray is tilted away from the waveguide.

As shown in Figure 12a, the RTR for four sample locations labeled as L1, L2, L3, and L4 within a microwave cavity illustrates the diversity of electromagnetic field distribution. The RTR dependency on sample location gives insight into the basic physic principle of microwave heating. It involves the formation of standing waves because of the incident and reflected electromagnetic waves, which form both hot and cold regions within the cavity. The non‐uniformity described above is mainly caused by the ratio of the cavity dimensions to wavelength, scaling together with the dielectric properties of the food samples that result in mismatches in impedance, affecting wave transmission and energy absorption. The discrepancies between experimental and calculated RTR values for chicken, and the consistency within the microwave oven position confirmed good model validation. The difference in RTR values noted between steamed chicken and steamed rice yields further insight. The difference is explained by rice dielectric properties. Rice contains more moisture and higher ionic concentration which translates to enhanced dielectric loss factor (*ε* ^″^) thereby allowing more efficient conversion of electromagnetic energy to thermal energy. Such a phenomenon obeys the equation *P* = 2*π*f *ε*_0_ *ε* ^″^ *E* ^2^, where *P* is the power absorbed per unit volume, *f* is the frequency,  *ε*_0_ is the permittivity of free space, *ε* ^″^ is the dielectric loss factor, and *E* is the electric field strength. Observation of food temperature change over time in Figure 12b shows that steamed rice at location L4 and steamed chicken breast at position L1 received the maximum heat. This was because the temperature was nearest to the waveguide where the electromagnetic field intensity is strongest. This case illustrates the inverse distance heating effect and efficiency from the energy source following the electromagnetic wave attenuation law. In Figure 13, the relationship of electric field strength to energy absorbed at L4 (135‐degree angle) demonstrates geometry related to heating efficiency, which is angle‐related. The angle of the position influences the electromagnetic field coupling efficiency because the waveguide aperture produces directional radiation patterns depending on the angle. The considerable difference between the two meals regarding energy absorbed at the location also incorporates factors of geometry and inherent differences in dielectric properties.

These results are important for commercial food processing businesses. Through automated tray positioning systems, industrial microwave units can apply the optimal positioning strategy of L4 for rice and L3 for chicken. Further scaling up needs focus on cavity design to allow multiple food trays while still using optimal electromagnetic field distribution. Through strategic positioning, industrial applications could achieve 15%–25% energy savings, which would be a significant operational cost savings for food processing plants with high throughput volumes.

**Figure 15:**
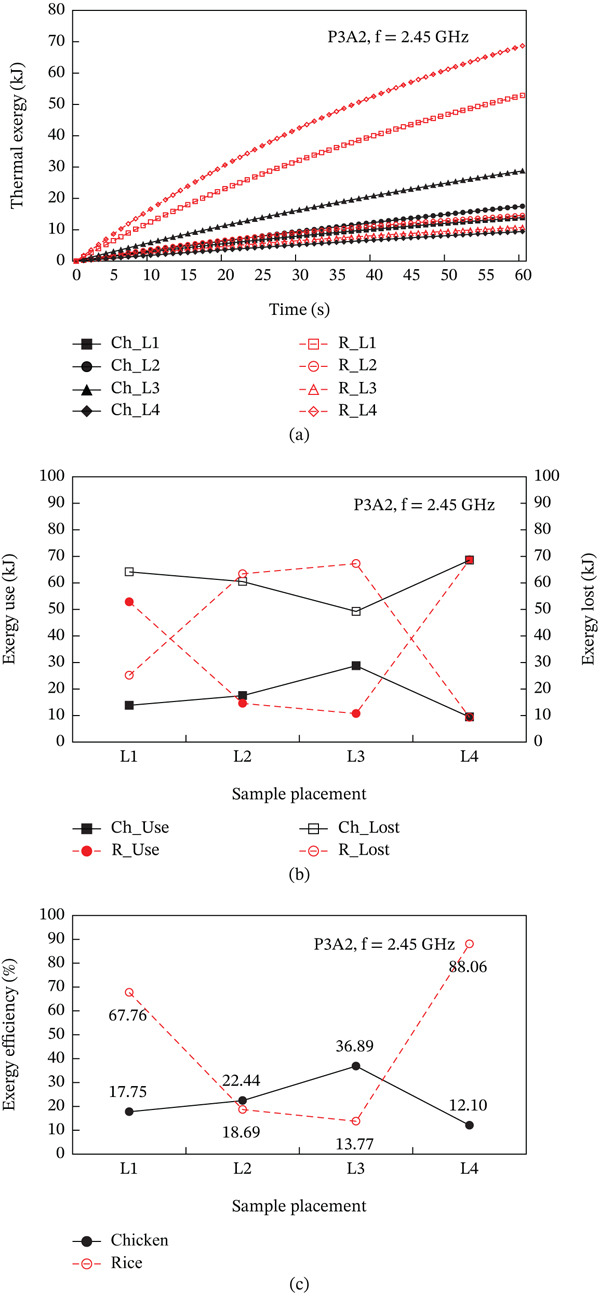
Influence of sample placement on the exergy transfer of ready‐to‐eat foods.

Safety concerning food hygiene is critical. This work demonstrates that proper settings can ensure minimum temperatures of 74°C for chicken products, thus adhering to FDA safety requirements for pathogens. The moisture distribution patterns (Figure 14) suggests food texture microbiological safety during strategic positioning. The nutrient‐loss risk for RTE foods is remarkably reduced due to shortened heating (60–90 s) compared with the conventional 5–8 min. Figure 15 shows the long‐term heating and exergy efficiency behavior. The thermal exergy analysis (Figure 15a) illustrates that extending heating past 60 s is inefficient, whereas 60–90 s provides optimal energy return. As indicated in Figure 15b, the energy efficiency utilization data confirms that steamed rice achieves maximum efficiency (88.06%) at L4 position while chicken breast optimization is angle dependent, peaking at L3 (36.89%). The sample placement interaction effect on energy efficiency stands as a major optimization criterion for stationary microwave systems. This research demonstrates that positioning significantly affects energy utilization efficiency, achieving a 20%–40% optimized strategic positioning enhancement without compromising food safety and quality. These conclusions underscore the enormous opportunities for reducing energy costs and enhancing efficiency in food processing through automated positioning systems in industrial microwave systems.

### 3.5. Optimal Operating Conditions

The combination of 1300 W microwave power, tray design A1 (semicircular compartments), and food placement angle L4 (135° to the waveguide) yielded the highest overall exergy efficiency. Under these conditions, exergy efficiency reached 85.0% for steamed rice and 50.7% for steamed chicken breast, with temperature uniformity maintained in the safe range of 45°C–60°C across both compartments. The optimal positions for non‐rotating ovens are L4 for rice and L3 for chicken to individually maximize efficiency, while the combined setting L4/A1/1300 W is the recommended configuration for reheating a mixed meal tray. These parameters provide actionable guidance for industrial microwave system operators and RTE food manufacturers seeking to optimize energy utilization while maintaining food safety standards.

## 4. Conclusion

This study focuses on microwave heating effectiveness from the perspective of the microwave power, tray design and placement with respect to the microwave, and their interactions in a single numerical model, which is new and different from other methods that focus on power balance or uniform heating. Rather, this is an integrated study that examines both heating and exergy transfer efficiency simultaneously.

Our results quantitatively show that the exergy efficiency max values for two main meal components, chicken breast and rice, were achieved at microwave powers of 800–1300 W with exergy efficiencies of 78.5% and 71.2%, respectively. The study confirmed that higher microwave power leads to higher electric field intensity and heating rate, also confirming improvements of temperature rise rates by 45%–60% within optimal power ranges. We found the heat‐mass transfer correlations which together show that exergy transfer efficiency dynamically regulates food dielectric constant (exergy transfer efficiency is controlled optimally by food’s dielectric constant). Container shape performs with significant influence on heating performance, especially that with rounded edges reduce electric field intensity by 23% and improves heat distribution uniformity by 35% as compared with rectangular containers. The research elaborated on industrial microwave system design and outlined tray positions for optimal microwave use: L4 for rice and L3 for chicken breast for non‐rotating microwaves. This allows industrial microwave manufacturers to refine chamber configurations and offers users scientifically validated instructions for reheating. The documented power to efficiency ratios established in the study can be used to form protocols for efficient energy use in cooking microwaves while maintaining the quality of food. Recommendations on container design enable manufacturers of RTE meals to improve product food packaging.

This study pioneered the understanding of high‐power microwave heating by developing and validating mathematical models of heat‐mass transfer in food matrices. For assessing these processes, we created a new type of exergy analysis specifically for food processing, allowing for a broader evaluation compared with conventional notions of heating efficiency. The study has taken a numerical modeling approach to capture electromagnetic fields and thermal gradients with moisture migration in multi‐component foods. These theoretical insights can be used as a basis for predictive modeling towards optimization in food microwave processing. Following this line of analysis, future work should incorporate other food matrices, such as liquids combined with solids and those with a more complex structure, to broaden their applicability. The incorporation of intelligent control systems for the heating elements provides a very interesting approach toward the evolution of adaptive microwave technologies that self‐modify parameters based on the food attributes. It is necessary to carry out scale‐up studies aimed at industrial application to test the results obtained in the laboratory and resolve possible difficulties of large‐scale applications. Further investigation into the work of real‐time monitoring systems could lead to the possibility of changing the heating parameters during microwave heating in real‐time. In summary, this study addressed three main research objectives: (1) the coupled heat–mass transfer mechanisms under high‐power microwave conditions were fully characterized: higher power levels (800–1300 W) increased heating rates by 45%–60%, and moisture redistribution rates reached up to 5.3448 mol m−3 s−1 for chicken in rectangular trays. (2) The exergy analysis revealed that electromagnetic‐to‐thermal conversion irreversibility and moisture evaporation are the dominant sources of exergy loss, with exergy efficiency ranging from 50.7% (chicken, A1/L4) to 85.0% (rice, A1/L4). (3) The optimal operating parameters were identified as 1300 W, tray A1, and placement L4 for a combined meal tray, with L4 for rice and L3 for chicken as individual optima in non‐rotating systems. Future research directions include the following: extension to heterogeneous food matrices including composite solid–liquid systems; integration of intelligent real‐time control algorithms that adapt power based on in‐situ temperature feedback; scale‐up validation studies in commercial‐scale conveyor microwave systems; and instrumental texture profiling (TPA) and proximate nutritional analysis to establish a comprehensive quality–efficiency correlation for industrially processed RTE meals.

## Nomenclature

**Latin Symbols**
C_*p*_
: specific heat capacity (J kg−1°C−1)
C_*w*_
: water concentration (mol m−3)
C_*w*,*i*_
: initial water concentration (mol m−3)
C_*w*,*s*_
: surface water concentration (mol m−3)
C_*e* *q* *u* *i*_
: equilibrium water concentration (mol m−3)
D_*w*_
: moisture diffusion coefficient (m2 s−1)
E^⟶^
: electric field intensity vector (V m−1)
ex: specific exergy (J kg−1)
${ex}_{evap}^{\prime }$
: rate of exergy transfer by evaporation (W)
Ex_*in*_
: input exergy (J)
Ex_*a* *b* *s*_
: absorbed exergy (J)
Ex_*l* *o* *s* *s*_
: specific exergy loss (J kg−1)
Ex_*r* *e* *f*_
: reflected exergy (J)
Ex_*t* *r* *a*_
: transmitted exergy (J)
f: microwave frequency (Hz)
h: convective heat transfer coefficient (W m−2°C−1)
k: thermal conductivity of food (W m−1°C−1)
k_0_
: complex wavenumber in vacuum (m−1)
$k_{m}^{\prime }$
: convective mass transfer coefficient (m s−1)
L: latent heat of water evaporation (= 2260 kJ kg−1)
m: mass of food sample (kg)
${\dot{m}}_{wv}$
: evaporation rate of water vapor (kg s−1)
P_*in*_
: input microwave power (W)
P_0_
: reference pressure (= 101.325 kPa)
Q_*a* *b* *s*_
: volumetric microwave heat generation (W m−3)
R_*w*_
: water evaporation source term (kg m−3 s−1)
T: temperature (°C)
T_*i*_
: initial temperature (°C)
T_0_
: reference (dead‐state) temperature (=25°C)
T_∞_
: ambient (far‐field) temperature (°C)
T_*p*_
: product (food) temperature during evaporation (K)
t: time (s)

**Greek Symbols**
*Δ* *t*
: time step (s)
*ε*_*r*_
: relative complex dielectric permittivity (*ε*_*r*_ = *ε*/*ε*_0_)
*ε* ^′^
: dielectric constant (real part of *ε*_*r*_) (–)
*ε* ^″^
: dielectric loss factor (imaginary part of *ε*_*r*_) (–)
*ε*_0_
: permittivity of free space (=8.854 × 10^−12^ F m^−1^)
*η*_ex_
: exergy efficiency (%)
*λ*_*w* *p*_
: latent heat of evaporation at product temperature (J kg^−^1)
*μ*_*r*_
: relative magnetic permeability (*μ*_*r*_ = *μ*/*μ*_0_)
*μ*_0_
: permeability of free space (= 4*π* × 10^−7^ H m^−1^)
ρ
: density of food (kg m^−3^)

**Abbreviations**
RTE: ready‐to‐eat
RTR: rate of temperature rise (°C s^−1^)
SD: standard deviation

## Funding

This study was supported by Rajamangala University of Technology Krungthep.

## Conflicts of Interest

The authors declare no conflicts of interest.

## Data Availability

The data that support the findings of this study are available on request from the corresponding author. The data are not publicly available due to privacy or ethical restrictions.
